# Image Quality, Radiation Dose, and Patient Comfort Associated with Wireless Sensors in Digital Radiography: A Systematic Review

**DOI:** 10.3390/dj12080267

**Published:** 2024-08-20

**Authors:** Carlos M. Ardila, Annie M. Vivares-Builes, Eliana Pineda-Vélez

**Affiliations:** 1 Department of Basic Sciences, Faculty of Dentistry, Universidad de Antioquia U de A, Medellín 050010, Colombia; 2 Research Department, Faculty of Dentistry, Institución Universitaria Visión de Las Américas, Medellín 050034, Colombia; anny.vivares@uam.edu.co (A.M.V.-B.); eliana.pineda@uam.edu.co (E.P.-V.)

**Keywords:** wireless technology, artificial intelligence, diagnostic imaging, endodontics, radiography

## Abstract

Radiography facilities face challenges with the positioning of digital radiography detectors. This study evaluates the image quality, radiation dose, and patient comfort associated with wireless sensors in digital radiography. A systematic exploration was performed across PubMed/MEDLINE, EMBASE, SCOPUS, Web of Science, and SCIELO. Nine papers met the eligibility criteria, including three observational studies with 111 patients, four in vitro experiments with 258 extracted human teeth, and two ex vivo investigations with 16 cadaver mandibles. All studies consistently reported high-quality images produced by wireless sensors. Two studies demonstrated the superiority of wireless sensors, one found comparable accuracy with conventional radiography, and another indicated similar image quality among the sensors. Both wireless and wired sensors significantly reduced radiation doses compared to conventional X-rays. The Visual Analog Scale (VAS) did not reveal a clear superiority of wireless over wired sensors, though both were generally less comfortable than traditional film. The wireless sensors consistently produce high-quality images, comparable to or superior to other digital devices. Both wireless and wired sensors significantly reduce radiation doses compared to conventional X-rays, emphasizing their safety and efficacy. Patient comfort levels vary, with neither sensor type showing clear superiority over the other, and both being less comfortable than traditional film.

## 1. Introduction

Intraoral periapical radiography is a commonly employed diagnostic imaging technique in dentistry, aimed at capturing detailed images of individual teeth and their surrounding structures [1,2,3]. These radiographs play a pivotal role in providing essential information for diagnosing various dental conditions and formulating appropriate treatment plans. Specifically in endodontics, intraoral periapical radiographs offer detailed images of root canals and surrounding anatomical elements, which are crucial for endodontists diagnosing infections, inflammations, or other pathologies within the root canal system [3]. Additionally, they aid in the identification of periapical lesions and are instrumental in effectively planning and executing root canal treatments [3,4]. Furthermore, radiographic assessments serve as a critical tool in the detection of proximal caries, an essential aspect of routine dental practice. The evaluation of digital systems often entails assessing their efficacy in diagnosing caries, thus highlighting the importance of evaluating their quality in clinical settings [5].

While conventional film technology continues to be widely utilized, there is a plethora of digital radiography alternatives currently available. These alternatives encompass a range of technologies that have advanced beyond conventional film, including digital sensors, phosphor plate systems, and portable digital X-ray devices [5,6]. These technologies offer several advantages, such as expedited image acquisition, improved image quality, reduced radiation exposure, and the convenience of digital storage and sharing. As a result, digital radiography has emerged as a prominent choice in contemporary dental and medical imaging practices [7,8].

In addition to direct digital and semi-direct digital systems, which utilize solid-state sensors or phosphor plate technology, respectively, other intraoral sensors include wireless and wired sensors. Wireless sensors transmit images wirelessly to a computer, providing flexibility during image acquisition. Conversely, wired sensors rely on physical cables for data transmission. Photostimulable phosphor sensors, also known as phosphor plate sensors, employ reusable imaging plates coated with a phosphor layer to capture X-ray energy, which are subsequently scanned to produce digital images. Each type of sensor possesses distinct features and workflows, catering to the various requirements of digital radiography [9].

Currently, radiography facilities face challenges related to positioning digital radiography detectors. To address these positioning requirements, wireless systems are available in various conformations, fluctuating from plain setups to completely computerized techniques. Compared to older, bulkier X-ray machines and systems, these mechanisms offer increased mobility and ease of relocation [10]. Furthermore, artificial intelligence is closely integrated with wireless sensors and digital radiology through its applications in image analysis, interpretation, and enhancement. Consequently, employing wireless sensors in digital radiography could play a crucial role in modern dental imaging. Acting as the image receptors in direct digital radiography systems, wireless sensors capture X-ray photons and swiftly convert them into electrical signals for immediate image acquisition [9,10]. The absence of cumbersome cables enables greater flexibility in sensor placement, which is particularly advantageous for capturing images in challenging anatomical areas. The wireless nature of the sensors simplifies both the setup and operation of the radiography system, reducing the need for complex wiring configurations and minimizing the risk of technical issues associated with wired connections [9,10,11].

Recognizing the importance of imaging as a diagnostic tool and noting the lack of a systematic evaluation regarding wireless sensors, it is crucial to evaluate the image quality of these sensors in digital radiography through a systematic review of studies conducted in this field. Furthermore, this investigation aims to evaluate radiation dose, and the comfort provided by intraoral mechanisms as additional objectives.

## 2. Materials and Methods

### 2.1. Protocol and Registration

This study followed a search methodology in accordance with the guidelines outlined by the preferred Reporting Items for Systematic Reviews and Meta-analyses (PRISMA) [12]. The protocol was officially recorded on PROSPERO and can be identified by the unique identifier CRD42024504397.

### 2.2. Suitability Criteria

The foundations of this study were laid by formulating a question in alignment with the Population, Intervention, Comparison, and Outcomes (PICO) framework:

P: studies assessing clinical images in digital radiography.

I: wireless sensors.

C: comparative control experiments.

O: image quality.

The secondary outcome variables included radiation dose, and the comfort provided by the intraoral mechanism.

The inclusion criteria were as follows: original articles of various designs or types published in any language, studies focusing on radiology or imaging utilizing wireless sensors, and investigations allowing for an evaluation of the performance of wireless sensors.

The exclusion criteria were as follows: conference proceedings, brief communications, abstracts, review articles, studies with unavailable or inaccessible full-text, and research missing indispensable aspects regarding the construction and method processes of wireless sensors.

### 2.3. Databases

A thorough search was conducted across multiple prominent scientific databases, including PubMed/MEDLINE, EMBASE, SCOPUS, Web of Science, and SCIELO, as well as Google Scholar for gray literature. The search spanned the entire history of these databases up to April 2024, without any language restrictions. Furthermore, additional relevant studies were identified by examining the reference lists and citations of all selected full-text articles, ensuring a comprehensive inclusion in the systematic review.

### 2.4. Search Strategy

The following terms were used: “wireless technology” OR “wireless sensors” OR “sensors” OR “dental digital radiography” OR “dental radiovisiography” OR “dental radiography” OR “diagnostic imaging” OR “digital technology” OR “dental radiography” OR “digital imaging” OR “bitewing radiography” OR “radiation dosage” AND “caries detection” AND “dental caries diagnosis” AND “dental pulp diseases” AND “root canal therapy” AND “periapical disease” AND “endodontics”.

These search strategies employ suitable syntax and operators for each database to retrieve pertinent articles relevant to the specified terms. Adjustments may be required depending on the specific search functionalities and syntax conventions of each database. Table 1 delineates the search strategies used for each designated database using the provided terms.

### 2.5. Study Selection

Two scholars separately reviewed titles and abstracts, and then thoroughly evaluated the full-text articles. They independently assessed the eligibility of the papers in duplicate, resolving any disagreements through discussion or consulting a third author if needed. To ensure consistency, inter-rater reliability was measured using the Kappa statistic, with a high agreement threshold of over 92%.

### 2.6. Data Collection

Two authors independently gathered data through individualized data extraction methods. Following this, a comparative analysis was performed to standardize the collected information. The data included specifics related to the operation of wireless sensors, encompassing essential characteristics such as the composition of materials used in their fabrication, as well as significant research outcomes including image quality, radiation dose, and comfort. The authors and the publication year were also documented.

### 2.7. Assessing Bias Risk and Study Quality in Individual Studies

We used the 16-item Quality Assessment Tool (QATSDD) [13] to evaluate the methodological quality of the included studies. This tool assesses 16 key aspects, including the following:

- A clear research framework and objectives;
- Well-defined study setting and sample;
- Robust data collection and analysis methods;
- Reliability and validity of measurement tools;
- Alignment between research question, data collection, and analysis;
- User involvement and critical discussion of strengths and limitations.

Each aspect is rated from 0 (insufficient detail) to 3 (fully provided). The total score, expressed as a percentage, represents the overall quality of the evidence.

### 2.8. Data Analysis

We collected mean differences and standard deviations from the studies, and considered meta-analysis when the data were consistent. No ethical approval was needed for this research.

## 3. Results

### 3.1. Study Selection

**Figure 1:**
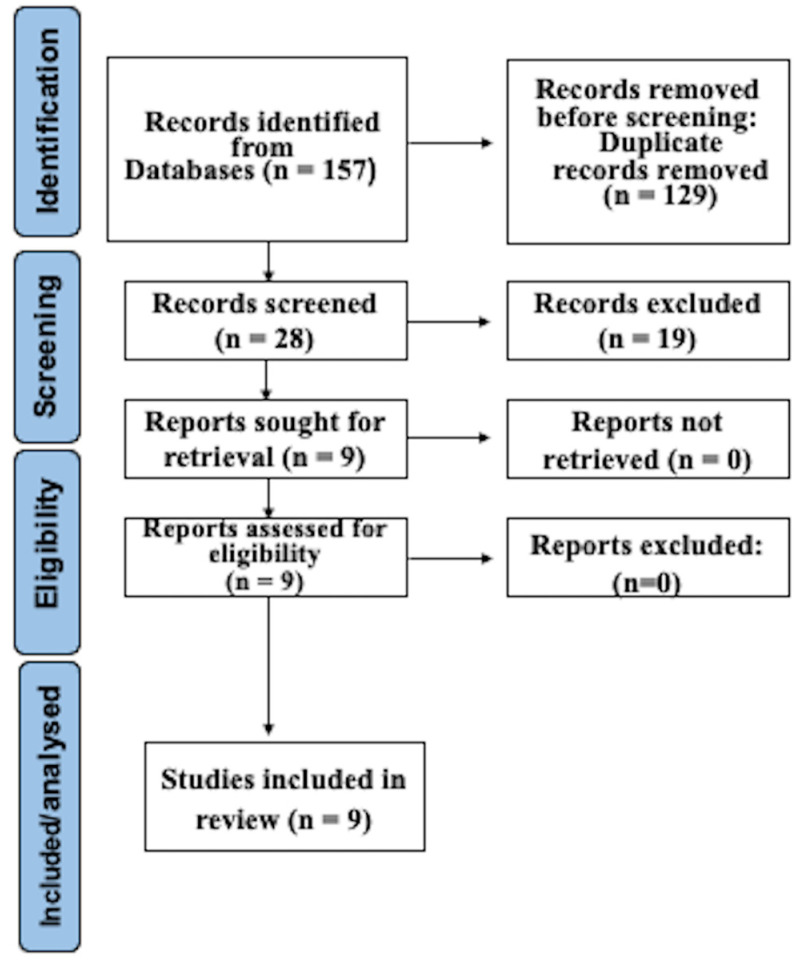
Prisma flowchart.

Following the search using the specified method, a total of 157 studies were identified. Following the elimination of duplicate entries and application of eligibility requirements, a thorough full-text evaluation was conducted on 28 remaining articles. Exclusion during the full-text review primarily occurred due to insufficient emphasis on wireless sensors and the absence of a control group. Ultimately, nine papers were selected for the systematic review after completing the final eligibility evaluation. The search process is illustrated in Figure 1, which outlines the step-by-step progression.

### 3.2. Descriptions of the Findings

Table 2 provides an overview of the key characteristics of the nine studies included in this study [14,15,16,17,18,19,20,21,22]. The analysis encompasses papers published from 2005 [21,22] to 2019 [14]. The nine included studies had different designs, encompassing three observational studies that evaluated 111 patients [15,17,21], four in vitro studies that experimented on 258 extracted human teeth [14,16,22], and two ex vivo studies that examined 16 human cadaver mandibles [19,20]. These studies were conducted in Europe [15,16,17], Brazil [14,15,16,17,18,19], the United States of America [20,22], and Asia [18,19,20,21]. These investigations evaluated images of root canals [18,20,21], periodontal ligaments [18,21], caries detection [14,15,16,19], and external root resorptions [18].

Table 3 demonstrates that all of the investigations consistently reported the capability of wireless sensors to produce high-quality images. However, while two studies demonstrated the superiority of wireless sensors [19,20], one study found a similar accuracy between conventional radiography and the wireless sensor but a better accuracy than the wired sensor [18], while the other investigation found similar image quality between the sensors [21]. Therefore, the quality of images from wireless sensors is reliably comparable or superior to other digital devices, with no instances of inferiority. Studies employing either wireless or wired sensors demonstrated a significantly reduced radiation dose compared to conventional X-rays [17,18,21]. In terms of patient-reported comfort with the sensors, the study utilizing the Visual Analog Scale (VAS) did not demonstrate the superiority of wireless over wired sensors. Nevertheless, both types of sensors were less comfortable than using film [21]. According to the analysis of variations in VAS scores among individual patients in another study, no significant differences were found between wireless sensors and other digital receptors [17].

In all the studies analyzed, a significant effort by the researchers was noted to control biases by standardizing the methodology for image acquisition, using the same radiological equipment, and employing independently calibrated operators, among other measures.

As observed in the analyzed studies, the technological and operating characteristics of wireless sensors used in digital radiography can vary depending on the specific model and manufacturer. However, some common features and characteristics include the utilization of technologies such as Bluetooth or Wi-Fi for transmitting data to the imaging system, eliminating the need for physical cable connections. Wireless sensors are available in various sizes to accommodate different imaging needs. The resolution of the sensor determines the level of detail captured in the images. Additionally, the sensor’s ability to capture low levels of radiation allows for reduced radiation doses during image acquisition. A broader dynamic range enables the sensor to capture a wide range of radiation intensities, ensuring an adequate representation of both dense and less dense tissues in the image. Wireless sensors demonstrate compatibility with standard radiographic equipment and imaging software. Manufacturers often provide dedicated image processing software, facilitating image enhancement, manipulation, and storage. The software includes tools for measurements and annotations, facilitating tasks such as making clinically appropriate referrals, for instance. Furthermore, wireless sensors are designed to be easily disinfected or equipped with disposable barriers, maintaining infection control standards.

### 3.3. Outcomes Analysis

Due to substantial variations in methodology and study design, this research did not allow for a meta-analysis to be carried out, precluding a quantitative synthesis of the results. Additionally, the seminal references were too diverse for an outcome assessment to be carried out, and the consideration of different wireless sensors with distinct designs further contributed to the methodological variation. Consequently, the focus shifted to a qualitative exploration.

### 3.4. Evaluation of Study Bias and Research Quality

All studies evaluated in this assessment achieved a quality score of at least 75%, indicating good quality (Table 4) [13]. While most studies had limitations, including inadequate sample size calculation and non-representative sampling, they nonetheless met the remaining quality criteria assessed by the evaluation method.

## 4. Discussion

This pioneering systematic review focuses exclusively on evaluating the quality of images from wireless sensors in digital radiography. Although the examined studies varied in their goals and methods, they unanimously demonstrated the effectiveness of the assessed wireless sensors, supported by robust validation protocols.

Image quality in digital radiography holds paramount importance in dentistry, facilitating precise diagnosis, optimal treatment planning, monitoring treatment progress, minimizing radiation exposure, enhancing communication, and educating patients. Investing in advanced imaging technologies is instrumental in elevating patient care standards and enhancing outcomes in the dental field.

In this systematic review, all the examined studies consistently affirm the capacity of wireless sensors to generate high-quality images. Notably, two studies showcase the superior performance of wireless sensors [19,20]. In contrast, one study identifies a comparable accuracy between conventional radiography and wireless sensors, with an improvement over wired sensors [18]. Meanwhile, other investigations observed a parity in image quality across the various sensors [14,15,16,21,22]. It has been observed that these variances could arise from the distinct technologies employed in generating the images [9,20]. For instance, the Schick CDR wireless system utilizes CMOS-APS (Complementary Metal-Oxide-Semiconductor-Active Pixel Sensor), while Gendex Visualix employs CCD (Charge-Coupled Device). Research by Athar et al. [20] revealed that CDR wireless sensors showed significantly improved accuracy in detecting key structures, outperforming CCD sensors. However, another study found that CCD sensors offered a marginally better spatial resolution (11 l/mm) compared to CDR wireless sensors (9 l/mm) [22]. Nevertheless, Kitagawa et al. [23] suggested that the CMOS sensor exhibited a superior performance compared to its CCD predecessor in representing cortical bone and root apices. The CCD detector excelled only in visualizing the root canal space, while no significant differences were found between CCD and Schick CMOS detectors in depicting periodontal ligament space or endodontic instruments. Both detectors produced radiographic images of similar overall quality. Notably, Okoro et al. [24] developed a portable dental imaging device, featuring a high-resolution CMOS sensor, fiber optic bundle, and integrated illumination, enabling precise root canal visualization during surgery. This innovative device successfully imaged various teeth with a resolution of approximately 48 μm and a 70-degree angular field-of-view.

While pixel size could influence outcomes, the similar pixel sizes of CCD and CDR wireless detectors (44 × 44 μm and 40 × 40 μm, respectively) suggest spatial resolution played a minor role. The 0.5 mm mean error difference, though statistically significant, may not be clinically significant [25]. Solid-state detectors demonstrated a superior performance compared to storage phosphor plates, likely due to distinct image acquisition mechanisms. Solid-state detectors employ pixel-based signal transmission, whereas storage phosphor plates use a screen-like process, potentially limiting spatial resolution due to light diffusion [20].

Remarkably, an additional investigation scrutinized in this systematic review noted that the conventional film and CCD wireless sensor outperformed the photostimulable phosphor (PSP) sensor in detecting external root resorption. The inferior performance of the PSP system may be due to various factors, including its lower resolution, complexities in scanning mechanisms, phosphor plate quality issues, an inadequate signal-to-noise ratio, and software limitations [18]. In contrast, digital radiography has been shown to surpass conventional imaging in detecting simulated external root resorption, with image magnification being a key contributing factor [26]. Similarly, another study found that enlarged digital images enhance the diagnosis of external root resorption compared to conventional radiography [27].

Given the significance of radiographic examinations as a crucial adjunct for detecting proximal caries, it is customary to assess the efficacy of digital systems in routine professional practice by evaluating their performance in the diagnosis of caries [14,16,17,18,19]. Haiter-Neto et al. [19] evaluated digital systems for proximal caries detection, using a wireless system with exposure times of 0.22 s for premolars and 0.26 s for molars, achieving a 64% accuracy rate. Notably, their results closely matched those of Melo et al. [14], who found optimal results at similar exposure times. Melo et al.‘s study revealed an intriguing pattern in the mean pixel values at 0.20 s, with two distinct behaviors potentially influenced by battery performance. Contrary to expectations, the mean pixel values did not consistently decrease with increasing exposure time, and the highest ROC (Receiver Operating Characteristic) value was found at the start of the second phase. While high-density images are ideal for caries diagnosis, Melo et al. identified a threshold density value for optimal results. However, their findings differ from Tsuchida et al. [21], who observed a stagnation point in the mean pixel values with increasing exposure doses. Further research with diverse sensors and batteries is needed to clarify these discrepancies [14].

Several methods assess sensor physical characteristics, including dose–response curves, Modulation Transfer Function (MTF), and Detective Quantum Efficiency (DQE). The dose–response curve evaluates sensitivity and contrast, while MTF measures resolution as a function of spatial frequency [21,28]. A study in this review found that the CDR wireless sensor’s sensitivity and contrast matched the wired CDR sensor’s [21], confirming previous research [29]. The DQE, representing X-ray absorption efficiency, was also assessed [21]. The measured DQEs for both sensors aligned with prior findings [30], showing a decrease with increasing exposure, consistent with other studies [31].

As delineated by the studies incorporated in this systematic review, using both wireless and wired sensors in digital radiography significantly contributes to a reduction in radiation dose compared to conventional X-rays, a fact previously substantiated [5,6,22]. Digital sensors exhibit an enhanced efficiency in converting X-ray signals into digital images compared to traditional film, necessitating shorter exposure times, and thereby diminishing the overall radiation dose. Both wireless and wired sensors often feature cutting-edge technologies, such as CMOS or CCD sensors, which excel at capturing X-ray energy and converting it into digital information. Furthermore, digital systems are frequently engineered to minimize scatter radiation, thereby aiding in the reduction in unnecessary radiation exposure to adjacent tissues.

To enhance comparability across different studies, it is essential to employ standardized criteria for assessing image quality and radiation dose. Future research should adopt established metrics such as the Modulation Transfer Function (MTF), Noise Power Spectrum (NPS), and Detective Quantum Efficiency (DQE). Additionally, standardized guidelines, like those provided by the American Association of Physicists in Medicine (AAPM) [32] and the International Commission on Radiological Protection (ICRP) [33], should be utilized to ensure consistent evaluation protocols. By adhering to these standardized criteria, researchers can achieve more reliable and comparable results, thereby advancing our understanding of image quality in wireless sensors and other digital radiography systems.

The wireless sensor offers the advantage of connecting peripherals within a 10-m radius, allowing for effortless setup throughout the X-ray room [18,21]. Additionally, the CDR wireless sensor’s image capture time is comparable to intraoral X-ray film and faster than the CDR sensor [18]. This means that, unlike wired sensors that may hinder mobility, the wireless sensor is easy to use like conventional intraoral X-ray film, enabling unrestricted movement during procedures [21].

In relation to the comfort perceived by patients when utilizing both wired and wireless sensors as well as conventional X-rays, one study examined in this systematic review indicated a higher level of comfort with conventional X-rays [21]. Two studies indicated no discernible differences in comfort between using wireless and wired sensors [17,21]. However, it was inferred that the wire primarily contributes to the sensation of discomfort [17]. Patient discomfort was assessed by Wenzel et al. [34] using a 100 mm VAS, yielding median scores of 20 mms for Digora and 32 mm for RVG. In contrast, Tsuchida et al.‘s [21] study found median VAS scores of 20 mm for wireless CDR, 16 mm for wired CDR, and 11 mm for film, suggesting differences in patient comfort among the imaging modalities. To further enhance patient comfort, it is crucial to address the ergonomic and design aspects of wireless sensors. Improving the form factor, size, and flexibility of these sensors can significantly reduce discomfort during use. Additionally, incorporating softer, biocompatible materials and rounded edges may enhance the overall patient experience. Collecting qualitative feedback from patients regarding their comfort and usability experiences can provide valuable insights for refining the design of wireless sensors. Such feedback can guide manufacturers in making ergonomic improvements that cater to patient needs and preferences, ultimately increasing the acceptability and effectiveness of these devices in clinical practice.

In addition to addressing comfort, it is essential to consider the benefits of innovative digital techniques in the diagnostic phase. The integration of advanced digital technologies can significantly enhance diagnostic accuracy and efficiency while reducing the radiological impact on patients. These systems leverage advanced image processing algorithms to enhance the visualization of anatomical structures, thereby aiding in more accurate diagnosis and treatment planning. Furthermore, the use of digital technologies enables a fully integrated workflow, enhancing clinical outcomes and patient safety. For instance, computer-assisted prosthetic planning and implant design with integrated digital bite registration, demonstrate the potential of digital workflows in improving diagnostic precision and reducing radiation exposure [35].

While wireless sensors confer various advantages in digital radiology, they are not without certain limitations. Some of these limitations encompass the following aspects. Wireless systems may be susceptible to interference from other electronic devices or environmental factors, potentially impacting signal quality and leading to artifacts in the images. The integration of wireless sensors with existing radiology equipment or picture archiving and communication systems could pose challenges, necessitating careful considerations of their compatibility with other components in the imaging workflow for seamless operation. The implementation of wireless technology might demand additional training for radiology staff to acquire proficiency in the utilization of new equipment and software, potentially affecting workflow during the initial adaptation period. Despite existing standards for digital radiography, variations in wireless technology standards may prevail. This lack of standardization can influence interoperability and restrict the interchangeability of wireless sensors across different systems. However, it is crucial to note that ongoing advancements in technology and research may gradually address some of these limitations. When considering the integration of wireless sensors into their practice, clinicians should carefully weigh and thoroughly assess the relevant factors.

This review possesses additional limitations associated with the limited number of studies that met the selection criteria, thereby impeding the presentation of more robust conclusions with a higher level of evidence. Nonetheless, these studies reported the capability of wireless sensors to generate high-quality images. Further investigations, particularly controlled clinical trials, are imperative to substantiate additional results.

A further challenge in this systematic review was the inability to perform a quantitative analysis, primarily due to the heterogeneity in study designs and methodologies. The reviewed studies differed in design, outcome assessment references, and sensor devices used; therefore, it is essential to identify and discuss potential sources of variability that contributed to the heterogeneity among the included studies. These sources of variability include differences in study design, population characteristics, sensor technology, outcome measures, and methodological approaches. The studies utilized a range of designs varied and could affect sensor performance and patient comfort. Additionally, the technological features and specifications of the wireless sensors differed, with variations in detector type (e.g., CMOS, CCD), pixel size, and spatial resolution impacting image quality, radiation dose, and patient comfort. The outcome measures employed to assess sensor performance, including image quality, radiation dose, and patient comfort, were diverse, leading to inconsistent reporting. Methodological differences, such as protocols for image acquisition, calibration, and analysis, further introduced bias and affected comparability. The heterogeneity among the included studies poses challenges in drawing definitive conclusions from this systematic review, complicating the synthesis of findings and potentially limiting the generalizability of results. Future research should aim to standardize study designs, harmonize outcome measures, include diverse populations, explore advanced sensor technologies, conduct longitudinal studies, and focus on ergonomic and design improvements to mitigate these sources of variability and enhance the quality and applicability of findings related to the use of wireless sensors in digital radiography.

Our review is particularly original in its comprehensive examination of wireless sensors’ performances in dental radiography, focusing not only on image quality and radiation dose but also on patient comfort. This holistic approach provides a multifaceted understanding of the benefits and limitations of wireless sensors, which is a relatively underexplored area in dental radiography research. This review addresses the gap in understanding how wireless sensors compare to both wired sensors and conventional radiographic methods across multiple dimensions, including image quality, radiation dose, and patient comfort. By synthesizing findings from various studies, our review advances current knowledge by highlighting the potential of wireless sensors to improve clinical outcomes while maintaining patient comfort and safety. Compared to the existing literature, our review stands out by systematically analyzing and summarizing data from multiple studies, providing a more comprehensive and nuanced understanding of wireless sensors’ performance. While previous reviews may have focused primarily on image quality or radiation dose, our review integrates these aspects with patient comfort and practical usability, offering a more complete picture of the technology’s impact in clinical settings. Our review provides new insights into the ergonomic and patient comfort aspects of wireless sensors, which have been relatively underexplored in previous research. Additionally, we present a detailed analysis of the variability in sensor performance due to different technological specifications and study designs, offering a clearer understanding of the factors that influence the effectiveness of wireless sensors in dental radiography.

Including larger and more diverse patient populations in future studies will help ensure that findings are more generally applicable. Expanding the scope of patient populations will contribute to a more comprehensive understanding of the performance of wireless sensors in digital radiography across different demographic groups. Such diversity will enhance the robustness and generalizability of the results, providing valuable insights into the efficacy of wireless sensors for a wider range of clinical scenarios. Furthermore, conducting longitudinal studies to assess the long-term outcomes and impacts of using wireless sensors in clinical practice is essential. These studies should evaluate the sustained performance, reliability, and clinical benefits of wireless sensors over extended periods. By monitoring the long-term efficacy and any potential issues that may arise, researchers can provide a more comprehensive understanding of the advantages and limitations of wireless sensors. This information is critical for clinicians to make informed decisions about integrating wireless sensor technology into routine practice and ensuring optimal patient care.

## 5. Conclusions

Wireless sensors consistently exhibit the ability to generate high-quality images, on par with or surpassing other digital devices. Both wireless and wired sensors offer markedly lower radiation doses compared to conventional X-rays, underscoring their safety and effectiveness in clinical settings. Although the comfort levels reported by patients may vary, neither sensor type demonstrates a distinct superiority over the other, with both generally being rated as less comfortable than traditional film. Hence, it is imperative to conduct comparative studies between conventional X-rays and wired sensors to gain a comprehensive understanding of the advantages and limitations associated with the utilization of wireless sensors in digital radiology.

## Figures and Tables

**Table 1:** Search strategies for each of the specified databases using the provided terms.

Database	Search Strategy
PuMed/MEDLINE	((“wireless technology” OR “wireless sensors” OR “sensors” OR “dental digital radiography” OR “dental radiovisiography” OR “dental radiography” OR “diagnostic imaging” OR “digital technology” OR “dental radiography” OR “digital imaging” OR “bitewing radiography” OR “radiation dosage”) AND “caries detection” AND “dental caries diagnosis” AND “dental pulp diseases” AND “root canal therapy” AND “periapical disease” AND “endodontics”)
Scopus	TITLE-ABS-KEY ((“wireless technology” OR “wireless sensors” OR “sensors” OR “dental digital radiography” OR “dental radiovisiography” OR “dental radiography” OR “diagnostic imaging” OR “digital technology” OR “dental radiography” OR “digital imaging” OR “bitewing radiography” OR “radiation dosage”) AND “caries detection” AND “dental caries diagnosis ” AND “dental pulp diseases” AND “root canal therapy” AND “periapical disease” AND “endodontics”)
Scielo	(“wireless technology” OR “wireless sensors” OR “sensors” OR “dental digital radiography” OR “dental radiovisiography” OR “dental radiography” OR “diagnostic imaging” OR “digital technology” OR “dental radiography” OR “digital imaging” OR “bitewing radiography” OR “radiation dosage”) AND “caries detection” AND “dental caries diagnosis” AND “dental pulp diseases” AND “root canal therapy” AND “periapical disease” AND “endodontics”
Embase	(‘wireless technology’/exp OR ‘wireless sensors’/exp OR ‘sensors’/exp OR ‘dental digital radiography’/exp OR ‘dental radiovisiography’/exp OR ‘dental radiography’/exp OR ‘diagnostic imaging’/exp OR ‘digital technology’/exp OR ‘dental radiography’/exp OR ‘digital imaging’/exp OR ‘bitewing radiography’/exp OR ‘radiation dosage’/exp) AND ‘caries detection’/exp AND ‘dental caries diagnosis’/exp AND ‘dental pulp diseases’/exp AND ‘root canal therapy’/exp AND ‘periapical disease’/exp AND ‘endodontics’/exp
Web of Science	TS = (“wireless technology” OR “wireless sensors” OR “sensors” OR “dental digital radiography” OR “dental radiovisiography” OR “dental radiography” OR “diagnostic imaging” OR “digital technology” OR “dental radiography” OR “digital imaging” OR “bitewing radiography” OR “radiation dosage”) AND TS = (“caries detection” AND “dental caries diagnosis” AND “dental pulp diseases” AND “root canal therapy” AND “periapical disease” AND “endodontics”)
Google Scholar	“wireless technology” OR “wireless sensors” OR “sensors” OR “dental digital radiography” OR “dental radiovisiography” OR “dental radiography” OR “diagnostic imaging” OR “digital technology” OR “dental radiography” OR “digital imaging” OR “bitewing radiography” OR “radiation dosage” AND “caries detection” AND “dental caries diagnosis” AND “dental pulp diseases” AND “root canal therapy” AND “periapical disease” AND “endodontics”

**Table 2:** Descriptive characteristics of the studies included.

Researchers and Publication Date	Nation	Study Design	Sample	Principal Purpose
Melo et al., 2019 [14]	Brazil	In vitro	40 teeth	Investigating the effect of varying exposure periods on caries diagnosis and image quality with a wireless procedure.
Hellén-Halme et al., 2013 [15]	Sweden	Observational	1 patient	Determining patient radiation levels resulting from 60 kV and 70 kV exposures for bitewing radiographs.
Hellén-Halme 2011 [16]	Sweden	In vitro	100 teeth	Comparing the influence of two distinct tube voltages on clinicians’ ability to identify proximal carious lesions in digital radiographs
Matzen et al., 2009 [17]	Denmark	Observational	110 patients	Comparing patient experience and image retake frequency between digital receptors and conventional film in wisdom teeth radiography.
Kamburoglu et al., 2008 [18]	Israel	Ex vivo	2 human cadaver mandibles	Comparing the diagnostic precision of conventional film, wireless digital sensors, and phosphor plates in identifying simulated external root resorption
Haiter-Neto et al., 2007 [19]	Brazil	In vitro	100 teeth	To evaluate the radiographic efficacy in detecting proximal carious lesions of two intraoral digital systems.
Athar et al., 2008 [20]	USA	Ex vivo	14 human cadaver mandibles	Assessing the measurement precision of a wireless image receptor relative to two alternative digital receptors for endodontic radiographic analysis.
Tsuchida et al., 2005 [21]	Japan	Observational	10 patients	An assessment was conducted on a wireless system, focusing on its physical attributes and operational simplicity.
Farman et al., 2005 [22]	USA	In vitro	18 teeth	Investigating the differences in spatial resolution, contrast visibility, and exposure tolerance among 18 dental X-ray detectors.

**Table 3:** Main outcomes associated with the selected articles.

Authors/ Publication Year	Intervention/ Control Group	Quality Image	Radiation	Comfort
Melo et al., 2019 [14]	Teeth underwent radiography utilizing a Schick CDR Wireless sensor across a range of exposure durations: 0.06, 0.10, 0.13, 0.16, 0.20, 0.25, 0.30, and 0.32 s. The scores were juxtaposed with histological sections of the teeth for comparison.	A 0.20-s exposure time produced consistent mean pixel values across both phases, revealing two distinct patterns. This decrease does not compromise image quality.	The Az values ranged from 0.53 to 0.62 across different exposure durations. Statistical evaluation indicated that the 0.25-s exposure period yielded the highest scores, significantly surpassing those obtained at 0.30 s and 0.35 s.	Not apply
Hellén-Halme et al., 2013 [15]	The distribution of absorbed doses resulting from two bitewing exposures was evaluated using a Planmeca DIXI2 and a CDR wireless sensor at tube voltages of 60 and 70 kV.	Reducing the dose to the patient did not compromise the image quality for the assessment of carious lesions.	Patient radiation dose is lowered when the tube voltage is decreased from 70 to 60 kV.	Not reported
Hellén-Halme 2011 [16]	Teeth underwent radiographic examination twice, employing wireless sensors, with tube voltages set at 60 and 70 kV, following a standardized protocol. Teeth histology was used as the definitive standard for assessment.	There was no statistically significant disparity in the precision of diagnosing approximal carious lesions between the two voltage settings.	A consensus among 5 observers deemed 70 kV radiographs superior for visualizing dentin lesions compared to 60 kV radiographs. The exposure durations at 70 kV were shorter than those at 60 kV.	Not apply
Matzen et al., 2009 [17]	Intraoral radiographs of both mandibular third molar areas were taken for each patient using two selected digital systems from a pool of five options.	Not reported	A significantly higher number of retakes were required with CDR-APS compared to CDR wireless (*p* < 0.019). Additionally, notable differences were observed between the PSP plates and wired sensors, with a higher retake rate seen when using wired sensors.	The variation in VAS scores within individual patients suggests that the wire is the main cause of discomfort, as no significant differences were found between CDR wireless and other digital receptors
Kamburoglu et al., 2008 [18]	Radiographs taken using conventional methods and digital technologies (CCD and PSP sensors)	The conventional film and wireless sensor produced a higher proportion of accurate readings compared to the PSP receptor.	Digital sensors can be utilized, offering the advantage of a lower radiation dose.	Not apply
Haiter-Neto et al., 2007 [19]	X-rays of cavity-free proximal surfaces were evaluated using Digora FMX, Digora Optime, Schick CDR, and wireless systems for comparison, with caries presence confirmed by histological examination.	Digora Optime and the wireless sensor exhibited significantly higher sensitivities compared to the other systems, whereas the wireless sensor demonstrated significantly higher specificity and positive predictive value than Digora Optime (*p* < 0.02).	The exposure durations for the chosen images were shorter for CDR and wireless sensors.	Not apply
Athar et al., 2008 [20]	Wireless image sensor was compared to two other types of digital image receptors.	The accuracy of raters in identifying structures of interest was significantly lower when using storage phosphor plates, whereas the wireless sensor showed the highest accuracy.	Not reported	Not apply
Tsuchida et al., 2005 [21]	A wireless sensor was compared to a wired sensor.	Both wired and wireless systems exhibited identical Modulation Transfer Functions, with comparable Detective Quantum Efficiency results.	The two sensors showed identical responses to radiation exposure, with gray levels decreasing linearly from 0 to 150 μGy and reaching maximum saturation at or above 150 μGy.	The visual analog scores assessing the discomfort associated with inserting and placing both the wireless and wired sensors in the mouth were similar.
Farman et al., 2005 [22]	Spatial resolution was tested with a 0.025 mm lead phantom grid (1.5–20 lp/mm). Contrast perception was evaluated using a 7 mm aluminum device featuring depth increments (0.1–0.9 mm) and a 1.5 mm defect. Relative exposure latitude was established through expert consensus, defining the lower limit as clear enamel-dentin junction visibility and the upper limit as pixel blooming or excessive burnout.	Eighteen detectors achieved contrast resolution of 0.2 mm or better through 7 mm aluminum, with top performance seen in wireless sensors and five additional detectors.	The lowest exposure ranges were observed in wireless sensors and four other systems.	Not apply

**Table 4:** Evaluation of Study Bias and Research Quality [13].

Investigation	Conditions Totally Fulfilled	Proportion of Fulfillment
Melo et al., 2019 [14]	14	87.5%
Hellén-Halme et al., 2013 [15]	13	81.3%
Hellén-Halme 2011 [16]	13	81.3%
Matzen et al., 2009 [17]	14	87.5%
Kamburoglu et al., 2008 [18]	12	75%
Haiter-Neto et al., 2007 [19]	13	81.3%
Athar et al., 2008 [20]	13	81.3%
Tsuchida et al., 2005 [21]	12	75%
Farman et al., 2005 [22]	13	81.3%

The 16-criterion instrument evaluates research quality, covering theoretical foundation, objectives and design, sampling and data collection, measurement tool validity, methodological alignment, analytical rigor, user involvement, critical self-assessment. This comprehensive framework ensures a thorough assessment of research methodology and quality.

## Data Availability

The data obtained in this review were pooled from the included investigations.
